# How *Shigella* Utilizes Ca^2+^ Jagged Edge Signals during Invasion of Epithelial Cells

**DOI:** 10.3389/fcimb.2016.00016

**Published:** 2016-02-10

**Authors:** Mariette Bonnet, Guy Tran Van Nhieu

**Affiliations:** ^1^Equipe Communication Intercellulaire et Infections Microbiennes, Centre de Recherche Interdisciplinaire en Biologie, Collège de FranceParis, France; ^2^Institut National de la Santé et de la Recherche Médicale U1050Paris, France; ^3^Centre National de la Recherche Scientifique, UMR7241Paris, France; ^4^MEMOLIFE Laboratory of Excellence and Paris Science LettreParis, France

**Keywords:** *Shigella* invasion, Ca^2+^ signaling, mitochondria-induced cell death, host cell survival, inflammation

## Abstract

*Shigella*, the causative agent of bacillary dysentery invades intestinal epithelial cells using a type III secretion system (T3SS). Through the injection of type III effectors, *Shigella* manipulates the actin cytoskeleton to induce its internalization in epithelial cells. At early invasion stages, *Shigella* induces atypical Ca^2+^ responses confined at entry sites allowing local cytoskeletal remodeling for bacteria engulfment. Global Ca^2+^ increase in the cell triggers the opening of connexin hemichannels at the plasma membrane that releases ATP in the extracellular milieu, favoring *Shigella* invasion and spreading through purinergic receptor signaling. During intracellular replication, *Shigella* regulates inflammatory and death pathways to disseminate within the epithelium. At later stages of infection, *Shigella* downregulates hemichannel opening and the release of extracellular ATP to dampen inflammatory signals. To avoid premature cell death, *Shigella* activates cell survival by upregulating the PI3K/Akt pathway and downregulating the levels of p53. Furthermore, *Shigella* interferes with pro-apoptotic caspases, and orients infected cells toward a slow necrotic cell death linked to mitochondrial Ca^2+^ overload. In this review, we will focus on the role of Ca^2+^ responses and their regulation by *Shigella* during the different stages of bacterial infection.

## Introduction

*Shigella*, the causative agent of bacillary dysentery, invades the colonic mucosa, where it induces a strong inflammatory response responsible for massive destruction of the epithelium (Ashida et al., 2015). *Shigella* crosses the intestinal barrier by transcytosis through M cells to reach the subepithelial tissue and invades colonocytes through the basolateral side (Sansonetti et al., 1996). Recent evidence suggests that *Shigella* can also invade colonocytes at the level of mouth crypts (Arena et al., 2015). Although less efficient, colonocyte invasion via the apical site may correspond to a discrete route enabling bacterial intracellular replication while dampening inflammatory responses.

*Shigella* invasion is a tightly regulated process involving the type III secretion system (T3SS) (Carayol and Tran Van Nhieu, 2013). By subverting cytoskeleton components, *Shigella* type III effectors trigger actin polymerization and membrane ruffling to induce its internalization by epithelial cell in a macropinocytic-like process (Valencia-Gallardo et al., 2015). Following invasion, *Shigella* escapes rapidly from the newly formed vacuole to reach the host cell cytosol, its replicative niche (Ray et al., 2010). The formation of an actin comet tail at one pole of the bacterium propels it in the cytoplasm and allows spread from cell to cell within the epithelium (Schroeder and Hilbi, 2008). In recent years, it has become clear that bacterial dissemination within the epithelium is critically dependent on the timely control of cell processes, such as autophagy, inflammatory signals and cell death pathways. While, as illustrated in this issue, various studies have described the involvement of type III effectors in these molecular processes, the role of second messengers has been relatively overlooked. Specifically, the role of Ca^2+^ signaling during pathogenesis is still poorly characterized, despite its importance and versatility. Here, we will review how *Shigella* hijacks Ca^2+^ signaling to promote invasiveness while tuning its deleterious effect to avoid premature cell death and inflammation.

**Figure 1:**
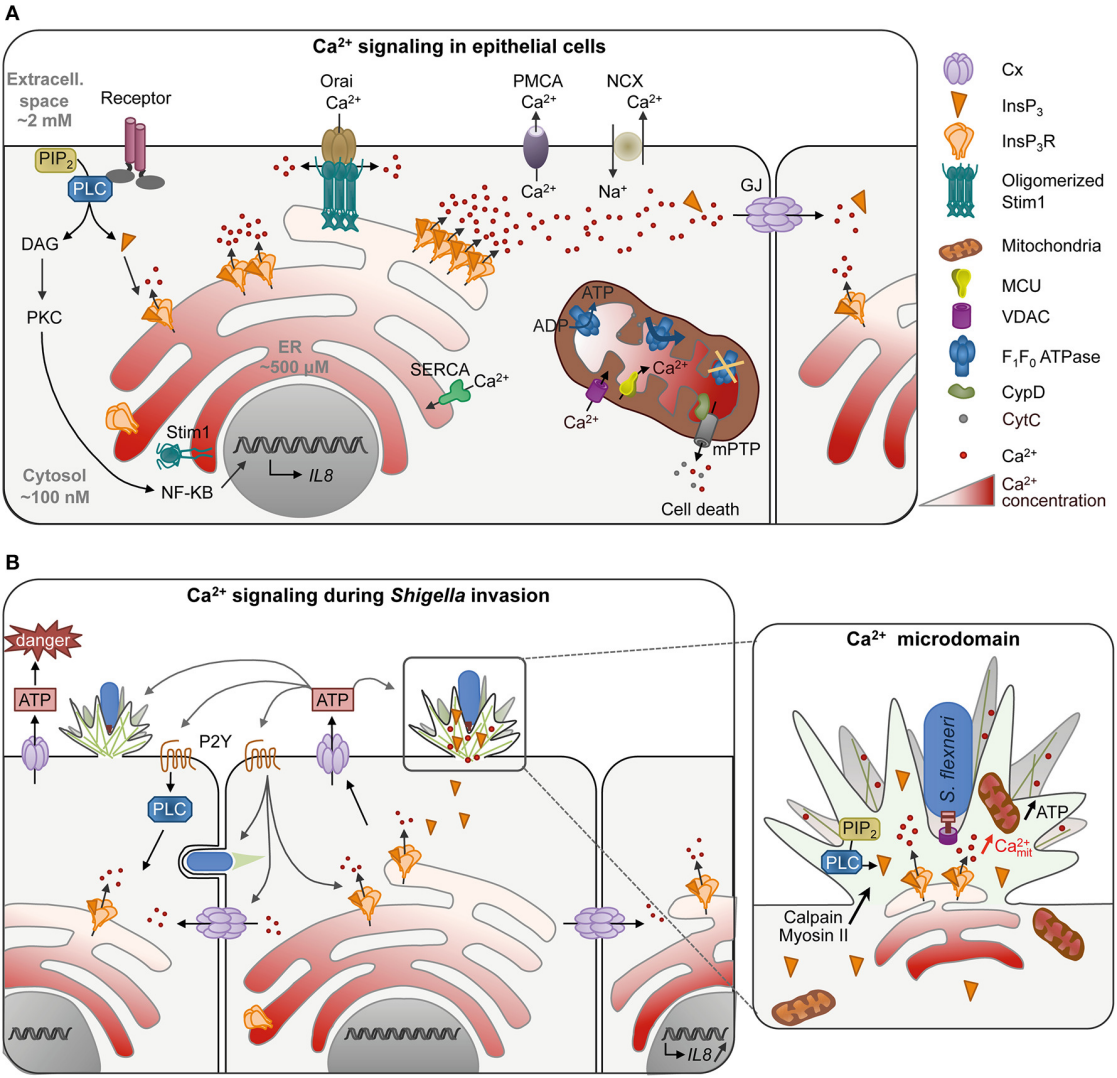
**Ca^**2+**^ signaling in epithelial cells in normal conditions (A) or during ***Shigella*** invasion (B)**. **(A)** Activation of cell receptor such as the G-protein coupled receptor at the plasma membrane (PM) stimulates the production of InsP_3_ by PLC hydrolysis of PIP_2_. Binding of InsP_3_ to its receptor InsP_3_R on the ER releases Ca^2+^ in the cytosol, translated into cellular processes such as transcription of cytokines by NF-κB. Low InsP_3_ production induces the opening of a single (blips) or a few InsP_3_R (puffs) resulting in Ca^2+^ microdomains, while high InsP_3_ levels generate global Ca^2+^ responses. Ca^2+^ waves can further propagate to the neighboring cells through gap junctions (GJ, 2 hexameric connexins apposed to each other forming a channel) to set up an intercellular wave. Ca^2+^ depletion in the ER induces the oligomerization of the Ca^2+^ level sensor protein STIM (stromal interaction molecule), which then interacts with the Ca^2+^ channel protein ORAI on the PM allowing Ca^2+^ influx. Restoration of basal cytosolic Ca^2+^ concentration is rapidly achieved by extrusion of Ca^2+^ across the PM by the Na^+^/Ca^2+^ exchanger or the Ca^2+^ ATPase PMCA or Ca^2+^ refilling into the ER by the Ca^2+^ ATPase SERCA. Mitochondria, often in vicinity to the ER, can buffer cytosolic Ca^2+^ by taking up Ca^2+^ through the mitochondrial outer membrane channel VDAC (Voltage-Dependent Anion Channel) and the Ca^2+^ uniporter MCU (Mitochondrial Ca^2+^ Uniporter) on the inner membrane. Ca^2+^ elevation in mitochondria activates mitochondrial functions such as the F1/F0 ATPsynthase. Ca^2+^ overload, however, inhibits ATP production and induces the opening of the mitochondrial permeability transition pore (mPTP) through CypD activation. mPTP releases small solutes such as Ca^2+^ and cytochrome c leading to cell death. **(B)** During invasion of epithelial cells, *Shigella* induces Ca^2+^ signaling by recruiting PLC at entry site. Due to the dense actin meshwork and recruitment of InsP_3_R in the foci, Ca^2+^ microdomains are confined in the entry site. Mitochondria trapped or in close vicinity to the entry site are activated with an increase of mitochondrial Ca^2+^ and produce more ATP necessary for actin foci formation. Ca^2+^ increase is required for efficient bacteria invasion through activation of Ca^2+^-dependent processes such as calpain and myosin II. *Shigella* invasion also induces global Ca^2+^ responses, which induce connexin (Cx) hemichannel opening at the PM, releasing ATP in the extracellular milieu. ATP signaling in turn increases the number of bacteria captured and entering the cell at a given invasion site, as well as bacterial invasion in the neighboring cells. It also increases global Ca^2+^ responses through purinergic reeptor signaling (P2Y). Global Ca^2+^ responses can further propagate to neighboring cells through gap junctions, leading to the transcription of IL-8 by bystander cells. *S. flexneri* is represented in blue with a Type III secretion system.

Ca^2+^ signaling is involved in virtually every cell biological processes. At basal state, the cytosolic Ca^2+^ concentration is low, in the hundreds of nanomolar range. In response to the activation of cell surface receptors, such as the G-protein coupled receptor (GPCR) at the plasma membrane (PM), the cytosolic Ca^2+^ concentration increases to reach a micromolar range (Figure 1A). Under physiological conditions, Ca^2+^ increases are transient and often oscillatory. The base for Ca^2+^ oscillations relies on an interplay between Ca^2+^ channels and pumps at the plasma and internal membranes. For example, Ca^2+^ increases can result from Ca^2+^ influx i.e., the uptake of extracellular Ca^2+^ by Ca^2+^ channels at the PM, or the release of Ca^2+^ from intracellular stores. In non-excitable cells, Ca^2+^ release is predominantly mediated by inositol-1,4,5-trisphophate receptors (InsP_3_Rs) on the endoplasmic reticulum (ER), which are InsP_3_-gated Ca^2+^ release channels. Following GPCR stimulation, InsP_3_ is generated by the hydrolysis of phosphatidylinositol 4,5-bisphosphate (PIP_2_) by phospholipase C (PLC). Increase in InsP_3_ levels triggers the opening of InsP_3_Rs and Ca^2+^ release. The consecutive Ca^2+^ depletion from the ER activates Ca^2+^ entry across the PM, a process called store-operated Ca^2+^ entry (SOCE) carried out by the interaction of the Ca^2+^ depletion sensor stromal interaction molecule (STIMs) on the ER and the Ca^2+^ release-activated Ca^2+^ channel protein ORAI on the PM. Restoration of basal cytosolic Ca^2+^ concentration is rapidly achieved by extrusion across the PM by the Na^+^/Ca^2+^ exchanger or the Ca^2+^ ATPase PMCA and Ca^2+^ refilling into the ER by the Ca^2+^ ATPase SERCA (Bootman, 2012, Figure 1A).

Sustained increases in high Ca^2+^ concentrations, however, lead to cell death due to mitochondrial Ca^2+^ overload (Calí et al., 2012). Upon Ca^2+^ release, mitochondria—associated or in close vicinity to ER membranes—take up Ca^2+^ via the mitochondrial outer membrane channel VDAC (Voltage-Dependent Anion Channel) and the Ca^2+^ uniporter MCU (Mitochondrial Ca^2+^ Uniporter). Increase in mitochondrial Ca^2+^ activates mitochondrial functions including ATP synthesis from oxidative phosphorylation. Because of their slow Ca^2+^ uptake rate, mitochondria also buffer cytosolic Ca^2+^ variations and play an important role in shaping physiological Ca^2+^ signals (Olson et al., 2012). Sustained mitochondrial Ca^2+^ accumulation, however, triggers the irreversible opening of the mitochondrial permeability transition pore (mPTP) mediated by cyclophilin D (CypD), influx of solutes into the mitochondrial matrix leading to the swelling of mitochondria and permeabilization of mitochondrial membranes. The release of the pro-apoptotic factor cytochrome C as well as ROS eventually leads to cell death by apoptosis or necrosis (Orrenius et al., 2015, Figure 1A).

Ca^2+^ oscillations can be explained by the bi-phasic regulation of InsP_3_Rs by cytosolic Ca^2+^, and vary in amplitude, frequency and duration of responses (Dupont et al., 2008). The cell's ability to trigger different types of oscillatory responses leading to the activation of various processes is the base of signal encoding during Ca^2+^ signaling (Smedler and Uhlén, 2014). Changes in the Ca^2+^ basal concentration are translated into distinct cellular processes by Ca^2+^-modulated proteins, in a process termed Ca^2+^ decoding (Carafoli, 2003). Global Ca^2+^ responses have been implicated in activation of the transcription factor NF-κB that regulates pro-inflammatory cytokines expression in response to bacterial invasion (Dolmetsch et al., 1997; Gewirtz et al., 2000). Global Ca^2+^ responses can further propagate to neighboring cells through gap junction channels composed of two hexameric connexins (Cx) (Leybaert and Sanderson, 2012, Figure 1A).

Besides these features associated with specific profiles of global Ca^2+^ responses, Ca^2+^ signals can also locally display spatial organization. Ca^2+^ microdomains have been described in response to weak InsP_3_ levels, leading to opening of a single (i.e., blips) or a small cluster of InsP_3_Rs (i.e., puffs) (LaFerla, 2002, Figure 1A). They are transient, short-lived Ca^2+^ responses that remain confined to a small cytoplasmic region at Ca^2+^ releasing sites. Ca^2+^ microdomains have been implicated in various processes involving cytoskeletal reorganization, such as phagocytosis (Nunes et al., 2012), chemotaxis (Tsai et al., 2015), or in filopodial dynamics through activation of calpain, a Ca^2+^-dependent cysteine protease (Kerstein et al., 2015).

## *Shigella* utilizes Ca^2+^ signaling during invasion of epithelial cells

Upon host cell invasion, *Shigella* induces local and global Ca^2+^ responses dependent on InsP_3_-mediated signaling with a pattern that differs significantly from classical agonist-induced Ca^2+^ response (Tran Van Nhieu et al., 2003, 2013, Figure 1B). Local Ca^2+^ responses are induced as early as 5 min after bacterial contact with epithelial cells, with a peak of responses at 15 min. Some of these local Ca^2+^ responses, called RATP for “Responses Associated with Trespassing Pathogens,” are highly atypical because they can last tens of seconds and are confined to the bacterial invasion site. They involve PLC-β1 and δ1 activated at *Shigella* entry sites, leading to the local accumulation of InsP_3_. The bacterial stimuli inducing PLCs' activation have not been identified yet, but depend on a functional T3SS. While injected effectors are dispensable, mutants defective for the translocon components IpaB or IpaC fail to recruit PLCs (Tran Van Nhieu et al., 2013). IpaB and IpaC are both predicted to contain coiled-coil domains and structural studies have shown that IpaB coiled-coil domain shared similarities with pore-forming toxins (Barta et al., 2012). Insertion of type III translocon in the PM may trigger Ca^2+^ signaling during *Shigella* invasion, since destabilization of the PM by pore-formin toxins is sufficient to activate PLCs, Ca^2+^ release and cytoskeletal reorganization (García-Sáez et al., 2011; Schwan et al., 2014). The particular duration of the local Ca^2+^ responses is due to the recruitment of InsP_3_Rs and the dense actin meshwork that restricts the diffusion of InsP_3_ at the entry site (Tran Van Nhieu et al., 2013, Figure 1B).

Shortly after the peak of long-lasting local Ca^2+^ responses, *Shigella* elicits isolated global Ca^2+^ responses with slow dynamics. These responses are not essential for *Shigella* entry but amplify it, through the release of ATP in the extracellular milieu by Cx-hemichannel opening at the PM (Tran Van Nhieu et al., 2003; Clair et al., 2008). Secreted ATP stimulates cellular functions in an autocrine or paracrine manner, such as Ca^2+^ signaling, through pathways involving P2Y purinergic receptors (Sáez and Leybaert, 2014; Figure 1B).

### Local Ca^2+^ signals are involved in *Shigella* entry

Inhibition of the local Ca^2+^ responses by the Ca^2+^ chelator BAPTA, as well as transfection of InsP_3_-5 phosphatase impaired *Shigella* induced actin foci formation, indicating that they participate in the invasion process. These local Ca^2+^ responses are likely to have multiple other implications during *Shigella* invasion. For example, calpain activation by cytosolic Ca^2+^ increase participates in the cytoskeleton reorganization necessary for bacterial entry (Bergounioux et al., 2012). Calpain targets several components regulating cytoskeleton reorganization. Some of these, such as the Src tyrosine kinase and cortactin, have been implicated in *Shigella* invasion, and local sustained Ca^2+^ increases could mediate their calpain-dependent regulation at invasions sites (Franco and Huttenlocher, 2005; Jeong et al., 2013). Myosin II, a Ca^2+^ dependent motor protein, has been implicated in *Salmonella* invasion into host cells, a process sharing similarities with *Shigella* invasion (Hänisch et al., 2011). Myosin II is recruited in *Shigella* entry foci (Clerc and Sansonetti, 1987). Ca^2+^ elevation in the foci could thus participate in actomyosin contraction during invasion (Valencia-Gallardo et al., 2015). Mitochondria are detected in the dense actin meshwork at bacterial invasion sites and display mitochondrial Ca^2+^ increase (Tran Van Nhieu et al., 2013). Mitochondrial activation through InsP_3_-mediated Ca^2+^ increase may lead to increased ATP production necessary for high-energy demanding processes such as actin filament elongation and myosin activation required for *Shigella* engulfment in the cell (Kuiper et al., 2008; Griffiths and Rutter, 2009).

The confinement of RATP at the entry site may also impact on other aspects linked to mitochondrial signals during the early stages of infection. The uptake of Ca^2+^ by mitochondria might also restrict Ca^2+^ increase to the entry site and block its diffusion to the remainder of the cell, as proposed in other systems (Rizzuto et al., 2012). Mitochondrial Ca^2+^ buffering has been implicated in various processes including vesicle secretion (Celsi et al., 2009). Since exocytic processes triggered during *Shigella* invasion participate in the subsequent lysis of the bacteria-containing vacuole (Mellouk et al., 2014), it is possible that mitochondrial Ca^2+^ also regulates later stages following *Shigella* invasion.

### Global Ca^2+^ responses enhance *Shigella* invasion and dissemination mediated by Cx signaling

Cx-mediated signaling favors both *Shigella* invasion and dissemination in epithelial cells (Tran Van Nhieu et al., 2013). The basis for enhancement of bacterial dissemination is not understood, but may involve processes similar to the ones described for the stimulation of invasion promoted by ATP released by Cx-hemichannels. Extracellular ATP stimulates bacterial capture by filopodia, a process that precedes *Shigella* invasion. The Erk MAP-kinase is stimulated by extracellular ATP and was found to control the elongation and retraction of filopodia, by regulating the rate of actin retrograde flow and the actin dynamics at the base of the filopodia in the cell cortex. ATP signaling thus increases the number of bacteria captured and entering the cell at a given site, as well as bacterial invasion in neighboring cells at early stage of infection (Romero et al., 2011, Figure 1B). While there is a lack of *in vivo* evidence for the relevance of increased bacterial invasion linked to extracellular ATP released from Cx-hemichanels, it is expected to play at initial local sites of bacterial breaching of the intestinal epithelium at the onset of the infectious process. Global Ca^2+^ increases could favor dissemination, either directly through the activation of myosin required for protrusion engulfment (Rathman et al., 2000), or by affecting junctional integrity (Lum and Morona, 2014). Cxs enable the propagation of Ca^2+^ responses among neighboring cells. Classically, these intercellular waves are described as a result of InsP_3_ diffusion through gap junctions, but can also result from signaling through Cx-hemichannels (Leybaert and Sanderson, 2012). In addition, such Cx-mediated intercellular Ca^2+^ waves stimulate IL-8 expression in bystander cells through cell-cell propagation of the activation signal of the NF-κB transcription factor and MAP-kinases, a process that may occur at later infectious stages. Thus, through Cx-mediated signaling, epithelial cells may amplify inflammatory signals and innate immunity against bacterial infection (Kasper et al., 2010). Cxs have been described to favor adherence and invasion of other pathogenic bacteria in epithelial cells, therefore facilitating bacterial infection *in vivo* (Velasquez Almonacid et al., 2009; Guttman et al., 2010; Puhar et al., 2013; Simpson et al., 2013).

## Ca^2+^-induced slow necrotic cell death

*Shigella* invasion is perceived as a genotoxic stress by epithelial cells, triggering the activation of the p53 pro-apoptotic signaling pathway at early stages of infection (Bergounioux et al., 2012, Figure 2). In addition, the pro-apoptotic growth arrest and DNA damage 45α (Gadd45α) factor is strongly induced, resulting in the induction of the intrinsic apoptosis pathway via loss of mitochondrial potential and caspase-9 activation (Lembo-Fazio et al., 2011). However, *Shigella* deploys different strategies to prevent rapid apoptotic death, including the degradation of p53 through Ca^2+^- and VirA dependent activation of calpain (Bergounioux et al., 2012), the activation of the PI3K/Akt pro-survival pathway by the type III effector IpgD and transcription of anti-apoptotic processes (Pendaries et al., 2006; Clark and Maurelli, 2007; Carneiro et al., 2009). *Shigella* also interferes with apoptotic processes by inhibiting the mitochondria-mediated release of cytochrome c through the pilus protein FimA (Sukumaran et al., 2010), and inhibiting caspases through the type III effectors Spa15 and OspC3 (Faherty and Maurelli, 2009; Kobayashi et al., 2013, Figure 2).

**Figure 2:**
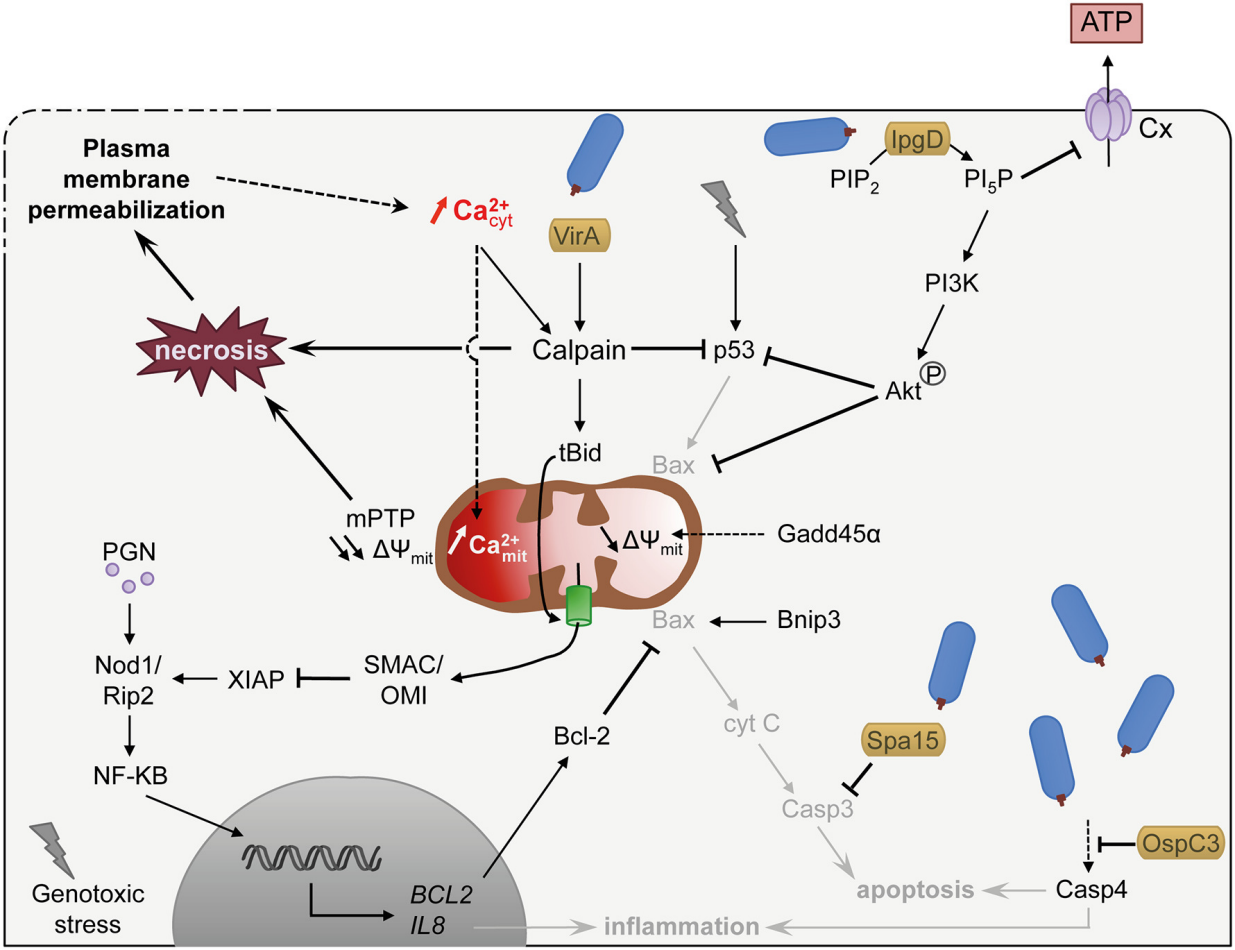
*****Shigella*** exploits Ca^**2+**^ signaling to delay cell death and to dampen inflammation**. *Shigella* invasion into host cell is perceived as genotoxic stress and induces apoptotic signaling mediated in part by the pro-apoptotic factor p53. *Shigella*, however, delays apoptotic cell death by different means: (i) promoting p53 degradation through Ca^2+^ increase and VirA dependent activation of calpain and IpgD-dependent activation of the PI3K/Akt survival pathway, (ii) maintaining mitochondria integrity by inhibiting Bax through Akt phosphorylation and NF-κB-dependent transcription activation of Bcl-2, (iii) inhibiting caspases by type III effectors. *Shigella* also dampens inflammation through IpgD-mediated closure of connexin hemichannel (Cx, in light violet) and calpain-mediated cleavage of Bid, which releases mitochondrial SMAC/OMI and antagonizes the inflammatory action of XIAP. Sustained cytosolic Ca^2+^ increase leads to mitochondrial Ca^2+^ overload, which induces a prolonged opening of the mPTP and ultimately necrosis. Necrosis is also promoted by pathologic activation of calpain. Altogether, the epithelial cell dies from a slow necrotic death. Plasma membrane permeabilization observed at later stages of infection might induce cytosolic Ca^2+^ increase, favoring necrotic cell death through mitochondrial overload. Dashed arrows indicate activation by an unknown mechanism. *S. flexneri* is drawn in blue. Type III effectors are labeled in yellow. Pathways hampered by *Shigella* are indicated in light gray. Lightning bolt represents genotoxic stress.

As a consequence, *Shigella*-infected epithelial cells do not die from apoptosis but from a slow necrotic death associated with PM permeabilization and increased cytosolic Ca^2+^ (Carneiro et al., 2009; Dupont et al., 2009, Figure 2). The cause for the increased cytosolic Ca^2+^ during cell infection by *Shigella* is not clearly established. It could be a direct result from Ca^2+^ influx linked to PM damage (Carneiro et al., 2009). Alternatively, it could result from the *Shigella*-induced activation of Bax/Bak or ER stress, shown in other studies to induce Ca^2+^ leakage from the ER (Distelhorst and Bootman, 2011; Tattoli et al., 2012; Tsalikis et al., 2015). The anti-apoptotic factor Bcl-2 is upregulated during *Shigella* invasion and delays *Shigella*-induced necrotic death (Carneiro et al., 2009). Bcl-2 is well characterized for its antagonistic function against MOMP (mitochondrial outer membrane permeability) and was proposed to counteract the pro-apoptotic role of Bnip3 during *Shigella* infection. Bcl-2, however, can also inhibit InsP_3_-mediated Ca^2+^ release and the mitochondrial Ca^2+^ overload (Distelhorst and Bootman, 2011), suggesting that it could also delay cell death through the modulation of Ca^2+^ responses.

Sustained increase in cytosolic Ca^2+^ favors the necrotic death of infected epithelial cells through diverse pathways. It leads to the pathological activation of calpain, committing the cells toward necrotic death (Łopatniuk and Witkowski, 2011; Bergounioux et al., 2012, Figure 2). It eventually also leads to increase in mitochondrial Ca^2+^, triggering mPTP opening mediated by CypD, resulting in loss of mitochondrial membrane potential, and eventually causes mitochondrial collapse, i.e., mitochondria swelling, loss of ATP production and ROS generation (Carneiro et al., 2009). The collapse of mitochondria depresses the levels of intracellular ATP below what is required for the execution of apoptosis, thus leading to necrotic death, for lack of ATP (Bernardi et al., 2015; Karch and Molkentin, 2015).

## Shigella manipulates Ca^2+^ signals to limit innate immunity and inflammation

As described elsewhere in this issue, *Shigella* utilizes different pathways to dampen innate immunity and inflammation. For example, the type III effector OspC3 inhibits caspase 4 activation, which would otherwise induce inflammatory cell death. In addition to a variety of type III effectors preventing the activation of NF-κB or MAP-kinases (Ashida et al., 2013), intracellular *Shigella* remodels its LPS into a poorly immunogenic form to better disseminate within the gut microenvironment (Paciello et al., 2013).

Ca^2+^-dependent calpain activation also dampens inflammatory signals during *Shigella* invasion. Calpain cleaves the pro-apoptotic BH3-only member of the Bcl-2 protein family BID, to trigger its translocation in mitochondria and the release of SMAC (second mitochondria-derived activator of caspases) and the mitochondrial serine protease Omi/HtrA2 (High-Temperature Requirement). SMAC and Omi/HtrA2 then antagonize the inflammatory action of XIAP (X-linked inhibitor of apoptosis protein; Andree et al., 2014, Figure 2).

Extracellular ATP acts as a local endogenous danger signal, triggering a protective inflammatory host response to eradicate pathogens (Bours et al., 2006). For example, extracellular ATP activates the differentiation of Th17 lymphocytes (Atarashi et al., 2008) and induces intracellular Ca^2+^ signaling leading to the activation of the NLRP3 (nucleotide-binding domain, leucine-rich-repeat-containing family, pyrin domain-containing 3) inflammasome (Murakami et al., 2012). ATP released by Ca^2+^-mediated opening of Cx-hemichannel during *Shigella* infection acts as a potent danger signal, triggering the release of cytokines oriented toward a proinflammatory Th17 cell response (Tran Van Nhieu et al., 2003; Puhar et al., 2013). At later stages following bacterial invasion, IpgD induces the closure of Cx-hemichannel thereby limiting ATP release in the extracellular medium and dampening inflammation (Puhar et al., 2013, Figure 2). The mechanism involved is not entirely clear but involves the IpgD-mediated production of phosphatidylinositol 5-phosphate (PI5P).

## Concluding remarks

We have discussed how *Shigella* uses Ca^2+^ signals from the onset of cell invasion to the death of infected cells. Local Ca^2+^ signals affect the dynamics of cytoskeletal reorganization to promote bacterial invasion. Global Ca^2+^ signals, usually associated with the induction of inflammatory signals, are not required for invasion and are involved at later stages of infection by *Shigella* to promote slow death. Between these two extremes of the cell infectious cycle and because of the versatility of Ca^2+^ signaling, processes key to *Shigella* virulence are likely to be regulated by Ca^2+^ signals, spatially and temporally regulated during the course of infection. The dual role of Ca^2+^-dependent calpain, involved in *Shigella* invasion and the orchestration of the death of infected cells illustrates this notion. How Ca^2+^ signals are generated and how they regulate the various processes associated with bacterial infection are key issues that cannot be ignored. Many pathogenic bacteria have been described to trigger Ca^2+^ responses during cell infection, involved in various key processes of infection such as bacterial adhesion, invasion, intracellular replication, or stimulating inflammation (Ruschkowski et al., 1992; Pace et al., 1993; Gewirtz et al., 2000; Asmat et al., 2014; Czyz et al., 2014; Jolly et al., 2014). The role of virulence factors has been studied through their catalytic activity or cellular compounds that they target. The role of bacterial factors on the regulation of second messenger such as InsP_3_, or ions such as Ca^2+^, has been seldom addressed, despite of their key role in fundamental cellular processes. The in-depth understanding of *Shigella*-induced signaling will implicate the integration of the diversion of the function of such second messengers during infection.

## Author contributions

All authors listed, have made substantial, direct and intellectual contribution to the work, and approved it for publication.

### Conflict of interest statement

The authors declare that the research was conducted in the absence of any commercial or financial relationships that could be construed as a potential conflict of interest.
